# Correction: Universal health coverage in the context of population ageing: catastrophic health expenditure and unmet need for healthcare

**DOI:** 10.1186/s13561-024-00495-6

**Published:** 2024-03-11

**Authors:** Shohei Okamoto, Mizuki Sata, Megumi Rosenberg, Natsuko Nakagoshi, Kazuki Kamimura, Kohei Komamura, Erika Kobayashi, Junko Sano, Yuzuki Hirazawa, Tomonori Okamura, Hiroyasu Iso

**Affiliations:** 1 Research Team for Social Participation and Healthy Aging, Tokyo Metropolitan Institute for Geriatrics and Gerontology, 35-2 Sakae-cho, Itabashi City, Tokyo, 1730015 Japan; 2 Institute for Global Health Policy Research, Bureau of International Health Cooperation, National Center for Global Health and Medicine, 1-21-1 Toyama, Shinjuku City, Tokyo Japan; 3 Research Center for Financial Gerontology, Keio University, 2-15-45 Mita, Minato City, Tokyo Japan; 4 Department of Preventive Medicine and Public Health, Keio University School of Medicine, 35 Shinanomachi, Shinjuku City, Tokyo Japan; 5 Clinical Epidemiology and Biostatistics, School of Medical Sciences, Faculty of Medicine and Health, Örebro University, Campus USÖ, Örebro, SE-701 82 Sweden; 6 World Health Organization Centre for Health Development, I.H.D. Centre Building, 9th Floor 7. 1-5-1 Wakinohama-Kaigandori, Chuo-ku, Kobe City, Hyogo Japan; 7 Hirao School of Management, Konan University, 8-33 Takamatsucho, Nishinomiya City, Hyogo Japan; 8 Faculty of Economics, Keio University, 2-15-45 Mita, Minato City, Tokyo Japan; 9 Tokyo Kasei Gakuin University, 22 Sanbancho, Chiyoda City, Tokyo Japan

**Correction to**: ***Health Econ Rev *14, 8 (2024)**

10.1186/s13561-023-00475-2

Following the publication of the original article [1], the license copyright has been corrected to CC BY 3.0 IGO instead of Creative Commons Attribution 4.0 International License. The full correct copyright line should read as below:

© World Health Organization 2024. **Open Access** This article is licensed under the terms of the Creative Commons Attribution 3.0 IGO License, which permits use, sharing, adaptation, distribution and reproduction in any medium or format, as long as you give appropriate credit to the World Health Organization, provide a link to the Creative Commons licence and indicate if changes were made. The use of the World Health Organization’s name, and the use of the World Health Organization’s logo, shall be subject to a separate written licence agreement between the World Health Organization and the user and is not authorized as part of this CC-IGO licence. Note that the link provided below includes additional terms and conditions of the licence. The images or other third party material in this article are included in the article’s Creative Commons licence, unless indicated otherwise in a credit line to the material. If material is not included in the article’s Creative Commons licence and your intended use is not permitted by statutory regulation or exceeds the permitted use, you will need to obtain permission directly from the copyright holder. To view a copy of this licence, visit http://creativecommons.org/licenses/by/3.0/igo/.

**Old Copyright Line**:

© World Health Organization 2024. Open Access This article is licensed under a Creative Commons Attribution 4.0 International License, which permits use, sharing, adaptation, distribution and reproduction in any medium or format, as long as you give appropriate credit to the original author(s) and the source, provide a link to the Creative Commons licence, and indicate if changes were made. The images or other third party material in this article are included in the article’s Creative Commons licence, unless indicated otherwise in a credit line to the material. If material is not included in the article’s Creative Commons licence and your intended use is not permitted by statutory regulation or exceeds the permitted use, you will need to obtain permission directly from the copyright holder. To view a copy of this licence, visit http://creativecommons.org/licenses/by/4.0/. The Creative Commons Public Domain Dedication waiver (http://creativecommons.org/publicdomain/zero/1.0/) applies to the data made available in this article, unless otherwise stated in a credit line to the data.

The original article [1] has been updated.
