# Supplementary material for: Universal health coverage in the context of population ageing: catastrophic health expenditure and unmet need for healthcare
Source: Health Econ Rev. 2024 Jan 30;14:8. doi: 10.1186/s13561-023-00475-2 (PMC10826197; doi:10.1186/s13561-023-00475-2)
Supplement: Supplementary file 1 — Supplementary Material 1: The Appendix note, tables, and figures are provided as the Supplementary material [file 13561_2023_475_MOESM1_ESM.docx]

**Supplementary material for**

**Universal health coverage in the context of population ageing:**

**Catastrophic health expenditure and unmet need for healthcare**

**Contents**

**Appendix note**

- Appendix note 1: Measures of financial protection and healthcare access

**Appendix tables**

- Appendix Table A-1. Catastrophic health expenditure comparing JHPS/KHPS and official government estimates using different thresholds
- Appendix Table A-2. Catastrophic health expenditure by age of household members
- Appendix Table A-3. Impoverishing health expenditure by age of household members
- Appendix Table A-4. Unmet healthcare need by age of respondents
- Appendix Table A-5. Heterogeneity by the presence of an older household member in the association between catastrophic health expenditure and food, culture/recreation, and social expenditure
- Appendix Table A-6. Financial consequences of catastrophic health expenditure

**Appendix figures**

- Appendix Figure A-1. Disparities in incomes and savings by the presence of older members in a household and CHE status

**References for Supplementary material**

**Appendix note 1. Measures of financial protection and healthcare access**

To ensure financial protection for UHC, two things are important. First, individuals should not suffer from undue financial hardship owing to out-of-pocket health spending; second, regardless of their demographic and socioeconomic status, individuals should not experience an unmet need for health services owing to financial barriers. CHE is a concept that helps measure the level of financial hardship caused by health services utilisation. CHE occurs when a household’s health expenditure exceeds a certain level of capacity, assuming that the level of health expenditure goes beyond one’s ability to pay, which may reduce the expenditure on other basic needs, such as education and food. A globally standardised measure of CHE, which is also used for measuring SDG Indicator 3.8.2, defines CHE as a household’s total health expenditure beyond the 10% and 25% thresholds of the total expenditure or income (1-5). To complement the CHE measure, some studies have used impoverishing health expenditure (IHE) as an indicator to estimate the difference in the poverty headcount with and without health expenditure to capture the relative impact of CHE on household finances (6).

Healthcare access is measured through healthcare utilisation or unmet need. Under the utilisation-based approach, it is measured as an individual’s actual use of healthcare services (7-10). Conversely, the measure of unmet need is an indicator of an individual’s lack of access to required health services. Many studies have measured unmet need based on self-assessed need for care and self-reported instances of foregone care in those situations (11-17). Both methods of assessing healthcare access can provide biased estimations owing to heterogeneous preferences over health and healthcare across demographic and socioeconomic groups in a health system with non-full access (11, 18). However, utilisation-based measures could even be biased in the assessment of equity in healthcare access, failing to consider differences in care-seeking preferences, even when no inequity exists (11). Although a measure for self-reported unmet need can have its limitations, self-assessed unmet need is related to poor individual health (11, 19) and population-level UHC service coverage (20). This suggests that self-reported unmet need can be useful for measuring healthcare access and may reflect true unmet need.

Appendix Table A-1. Catastrophic health expenditure comparing JHPS/KHPS and official government estimates using different thresholds

|  | JHPS/KHPS | | | National estimates | |
| --- | --- | --- | --- | --- | --- |
| year | 10% threshold | 25% threshold | N | 10% threshold | 25% threshold |
| 2004 | 8.9% | 1.4% | 3,433 | - | - |
| 2005 | 9.8% | 1.1% | 3,003 | - | - |
| 2006 | 10.4% | 1.5% | 2,758 | - | - |
| 2007 | 12.4% | 1.6% | 3,824 | - | - |
| 2008 | 11.2% | 1.4% | 3,500 | - | - |
| 2009 | 8.6% | 1.7% | 6,708 | - | - |
| 2010 | 9.7% | 1.3% | 6,067 | 9.1% | 1.6% |
| 2011 | 9.5% | 1.9% | 5,751 | 9.2% | 1.6% |
| 2012 | 10.3% | 2.0% | 6,215 | 9.3% | 1.7% |
| 2013 | 9.3% | 1.1% | 5,775 | 9.1% | 1.6% |
| 2014 | 9.5% | 1.8% | 5,251 | 9.1% | 1.6% |
| 2015 | 8.1% | 2.2% | 4,905 | 9.2% | 1.6% |
| 2016 | 9.4% | 1.6% | 4,617 | 9.4% | 1.6% |
| 2017 | 8.6% | 1.4% | 4,221 | 9.6% | 1.6% |
| 2018 | 8.6% | 1.6% | 3,861 | 9.7% | 1.7% |
| 2019 | 8.0% | 2.0% | 5,272 | 10.5% | 1.9% |
| 2020 | 9.9% | 2.1% | 3,368 | 10.9% | 1.8% |

Note: Weighted by cross-sectional and longitudinal weights; Official government estimates were obtained from <https://www.mofa.go.jp/mofaj/gaiko/oda/sdgs/statistics/goal3.html>

Appendix Table A-2. Catastrophic health expenditure by households with different age structures

|  | All 64 or younger | | | At least one person, 65 or older | | |
| --- | --- | --- | --- | --- | --- | --- |
| year | 10% threshold | 25% threshold | N | 10% threshold | 25% threshold | N |
| 2004 | 6.9% | 1.2% | 2,814 | 19.5% | 2.3% | 619 |
| 2005 | 8.5% | 0.8% | 2,424 | 15.5% | 2.2% | 579 |
| 2006 | 8.2% | 0.9% | 2,148 | 20.2% | 4.2% | 610 |
| 2007 | 9.9% | 1.0% | 2,986 | 22.9% | 4.1% | 838 |
| 2008 | 9.9% | 1.1% | 2,655 | 15.8% | 2.6% | 845 |
| 2009 | 6.0% | 1.1% | 4,761 | 14.0% | 3.1% | 1,947 |
| 2010 | 6.7% | 0.7% | 4,214 | 15.2% | 2.4% | 1,853 |
| 2011 | 7.3% | 1.4% | 3,922 | 13.8% | 2.7% | 1,829 |
| 2012 | 8.0% | 1.3% | 4,206 | 14.7% | 3.6% | 2,009 |
| 2013 | 6.5% | 0.7% | 3,779 | 14.7% | 1.9% | 1,996 |
| 2014 | 6.5% | 0.9% | 3,285 | 14.6% | 3.4% | 1,966 |
| 2015 | 5.4% | 1.5% | 2,968 | 12.8% | 3.3% | 1,937 |
| 2016 | 5.2% | 0.8% | 2,697 | 16.2% | 2.8% | 1,920 |
| 2017 | 6.3% | 1.0% | 2,729 | 14.8% | 2.5% | 1,492 |
| 2018 | 6.4% | 1.1% | 2,421 | 14.1% | 2.8% | 1,440 |
| 2019 | 5.5% | 1.0% | 3,383 | 10.9% | 3.1% | 1,889 |
| 2020 | 8.0% | 1.7% | 1,986 | 14.2% | 3.0% | 1,382 |

Note: Households were categorised by the age of the oldest co-residing family members, including respondents themselves; Weighted by cross-sectional and longitudinal weights.

Appendix Table A-3. Impoverishing health spending by households with different age structures

|  | Total | | All 64 or younger | | At least one person, 65 or older | |
| --- | --- | --- | --- | --- | --- | --- |
| year | Incidence | N | Incidence | N | Incidence | N |
| 2004 | 0.7% | 3,340 | 0.6% | 2,734 | 1.2% | 606 |
| 2005 | 0.9% | 2,804 | 0.5% | 2,288 | 2.5% | 516 |
| 2006 | 1.3% | 2,570 | 0.7% | 2,015 | 4.0% | 555 |
| 2007 | 1.1% | 3,557 | 0.7% | 2,777 | 2.7% | 780 |
| 2008 | 0.8% | 3,289 | 0.6% | 2,504 | 1.5% | 785 |
| 2009 | 1.8% | 6,198 | 1.3% | 4,438 | 3.0% | 1,760 |
| 2010 | 1.1% | 5,700 | 0.5% | 3,981 | 2.1% | 1,719 |
| 2011 | 1.0% | 5,377 | 0.3% | 3,692 | 2.2% | 1,685 |
| 2012 | 1.2% | 5,813 | 0.7% | 3,975 | 2.2% | 1,838 |
| 2013 | 0.8% | 5,416 | 0.6% | 3,589 | 1.3% | 1,827 |
| 2014 | 0.9% | 4,972 | 0.3% | 3,146 | 1.8% | 1,826 |
| 2015 | 1.0% | 4,663 | 0.7% | 2,862 | 1.3% | 1,801 |
| 2016 | 0.8% | 4,353 | 0.2% | 2,574 | 1.8% | 1,779 |
| 2017 | 1.1% | 3,873 | 0.6% | 2,547 | 2.5% | 1,326 |
| 2018 | 1.1% | 3,674 | 0.6% | 2,341 | 2.5% | 1,333 |
| 2019 | - | - | - | - | - | - |
| 2020 | - | - | - | - | - | - |

Note: Weighted by cross-sectional and longitudinal weights; In 2019 and 2020, the poverty line was not imputed because the poverty line after 2018 was not proved by the national government.

Appendix Table A-4. Unmet health care need by the age of respondent

|  | Total | | 64 or younger | | 65 or older | |
| --- | --- | --- | --- | --- | --- | --- |
| year | Prevalence | N | Prevalence | N | Prevalence | N |
| 2004 | - | - | - | - | - | - |
| 2005 | 11.3% | 2,157 | 12.6% | 1,667 | 6.5% | 490 |
| 2006 | 13.1% | 1,963 | 15.5% | 1,476 | 5.6% | 487 |
| 2007 | - | - | - | - | - | - |
| 2008 | 10.0% | 2,414 | 10.9% | 1,728 | 7.5% | 686 |
| 2009 | 9.2% | 2,333 | 10.0% | 1,640 | 7.0% | 693 |
| 2010 | 10.0% | 2,224 | 11.4% | 1,508 | 7.0% | 716 |
| 2011 | 10.2% | 2,101 | 12.2% | 1,385 | 6.5% | 716 |
| 2012 | 9.0% | 2,698 | 9.3% | 1,782 | 8.6% | 916 |
| 2013 | 7.8% | 2,494 | 9.3% | 1,575 | 5.3% | 919 |
| 2014 | 8.7% | 4,075 | 11.7% | 2,345 | 4.7% | 1,730 |
| 2015 | 9.6% | 3,861 | 12.8% | 2,121 | 5.5% | 1,740 |
| 2016 | 9.3% | 3,631 | 11.3% | 1,917 | 7.0% | 1,714 |
| 2017 | 7.2% | 3,397 | 9.0% | 1,992 | 3.6% | 1,405 |
| 2018 | 5.2% | 3,431 | 6.5% | 2,000 | 2.6% | 1,431 |
| 2019 | 5.1% | 4,781 | 7.1% | 2,884 | 3.4% | 1,897 |
| 2020 | 4.7% | 2,997 | 6.2% | 1,650 | 1.8% | 1,347 |

Note: *Unmet needs* excludes those who did not experience forgone care because they were healthy; In 2004 and 2007, the question on unmet health need was not asked; Weighted by cross-sectional and longitudinal weights; Age group categorisation is based on age of survey respondents.

Appendix Table A-5. Heterogeneity by the presence of an older household member in the association between catastrophic health expenditure and food, culture/recreation, and social expenditure

|  | Expenditures | | | | | | | | | | | | | | | |
| --- | --- | --- | --- | --- | --- | --- | --- | --- | --- | --- | --- | --- | --- | --- | --- | --- |
|  | Food | | | | Culture and recreation | | | | Social | | | | Education | | | |
| 65+ y/o member in a household | 0.01** | 0.01** | 0.01** | 0.01** | 0.00 | 0.00# | 0.00# | 0.00# | -0.00 | -0.00 | -0.00 | -0.00 | -0.00 | -0.00** | -0.00 | -0.00# |
|  | (0.00) | (0.00) | (0.00) | (0.00) | (0.00) | (0.00) | (0.00) | (0.00) | (0.00) | (0.00) | (0.00) | (0.00) | (0.00) | (0.00) | (0.00) | (0.00) |
| CHE10 | -0.03** | -0.03** |  |  | -0.01** | -0.01** |  |  | -0.02** | -0.02** |  |  | -0.00** | -0.01** |  |  |
|  | (0.00) | (0.00) |  |  | (0.00) | (0.00) |  |  | (0.00) | (0.00) |  |  | (0.00) | (0.00) |  |  |
| 65+ y/o in a household * CHE10 |  | -0.01** |  |  |  | -0.00 |  |  |  | -0.01* |  |  |  | 0.01** |  |  |
|  |  | (0.00) |  |  |  | (0.00) |  |  |  | (0.00) |  |  |  | (0.00) |  |  |
| Health expenditure |  |  | -0.23** | -0.20** |  |  | -0.07** | -0.07** |  |  | -0.12** | -0.10** |  |  | -0.03** | -0.05** |
|  |  |  | (0.01) | (0.01) |  |  | (0.01) | (0.01) |  |  | (0.01) | (0.01) |  |  | (0.00) | (0.00) |
| 65+ y/o in a household * Health expenditure |  |  |  | -0.08** |  |  |  | -0.00 |  |  |  | -0.05** |  |  |  | 0.05** |
|  |  |  |  | (0.02) |  |  |  | (0.01) |  |  |  | (0.01) |  |  |  | (0.00) |
| Individuals | 7,892 | | | | 7,898 | | | | 7,898 | | | | 7,898 | | | |
| Observations | 65,521 | | | | 65,564 | | | | 65,564 | | | | 65,564 | | | |

Note: CHE10 denotes catastrophic health expenditure at a 10% threshold; Each expenditure (% of total consumption) is transformed by the inverse hyperbolic sine transformation; Health expenditure (% of total consumption) is mean-centralised and transformed by the inverse hyperbolic sine transformation; Estimates by fixed-effects linear models; ** p<0.01, * p<0.05, ^#^ p<0.10; Values are coefficients with cluster-robust standard errors in parentheses; Controlled for income, savings, education of household head, employment status of household head, house ownership, household size, individual fixed-effects, and city-by-year fixed-effects; Weighted by longitudinal weights to address for attrition bias; singleton observations are not used for estimations.

Appendix Table A-6. Financial consequences of catastrophic health expenditure

|  | Income | | | | Savings | | | |
| --- | --- | --- | --- | --- | --- | --- | --- | --- |
| CHE10[t-1] | -0.01 | -0.02* |  |  | 0.02 | 0.04 |  |  |
|  | (0.01) | (0.01) |  |  | (0.03) | (0.04) |  |  |
| 65+ y/o member in a household | -0.03** | -0.04** | -0.03** | -0.03** | 0.04 | 0.05 | 0.04 | 0.04 |
|  | (0.01) | (0.01) | (0.01) | (0.01) | (0.04) | (0.04) | (0.04) | (0.04) |
| CHE10[t-1] * 65+ y/o member in a household |  | 0.03# |  |  |  | -0.05 |  |  |
|  |  | (0.01) |  |  |  | (0.05) |  |  |
| Health expenditure[t-1] |  |  | -0.05 | -0.12# |  |  | 0.08 | 0.29 |
|  |  |  | (0.04) | (0.06) |  |  | (0.14) | (0.19) |
| Health expenditure[t-1] * 65+ y/o member in a household |  |  |  | 0.15# |  |  |  | -0.45# |
|  |  |  |  | (0.08) |  |  |  | (0.28) |
| Household head being university graduate or higher | 0.07* | 0.07* | 0.07* | 0.07* | -0.04 | -0.04 | -0.04 | -0.04 |
|  | (0.03) | (0.03) | (0.03) | (0.03) | (0.08) | (0.08) | (0.08) | (0.08) |
| Household head being in paid work | 0.21** | 0.21** | 0.21** | 0.21** | -0.02 | -0.02 | -0.02 | -0.02 |
|  | (0.02) | (0.02) | (0.02) | (0.02) | (0.04) | (0.04) | (0.04) | (0.04) |
| House ownership | 0.11** | 0.11** | 0.11** | 0.11** | -0.06 | -0.06 | -0.06 | -0.06 |
|  | (0.02) | (0.02) | (0.02) | (0.02) | (0.08) | (0.08) | (0.08) | (0.08) |
| Household size | -0.19** | -0.19** | -0.19** | -0.19** | -0.35** | -0.35** | -0.35** | -0.35** |
|  | (0.02) | (0.02) | (0.02) | (0.02) | (0.05) | (0.05) | (0.05) | (0.05) |
| Individual-FE | Yes | Yes | Yes | Yes | Yes | Yes | Yes | Yes |
| City-by-Year-FE | Yes | Yes | Yes | Yes | Yes | Yes | Yes | Yes |
| Constant | 6.41** | 6.41** | 6.40** | 6.40** | 5.49** | 5.49** | 5.50** | 5.50** |
|  | (0.03) | (0.03) | (0.03) | (0.03) | (0.09) | (0.09) | (0.09) | (0.09) |
| Individuals | 7,010 | | | | 7,033 | | | |
| Observations | 55,233 | | | | 55,409 | | | |

Note: CHE10 denotes catastrophic health expenditure at a 10% threshold; Health expenditure (% of total consumption) is mean-centralised and transformed by the inverse hyperbolic sine transformation; Estimates by fixed-effects linear models; ** p<0.01, * p<0.05, ^#^ p<0.1; Values are coefficients with cluster-robust standard errors in parentheses; FE represents fixed-effects; Income and savings are equivalised by household size and transformed by the inverse hyperbolic sine transformation; Household size represents the log-transformed number of household members; Weighted by longitudinal weights to address for attrition bias; singleton observations are not used for estimations.

Appendix Figure A-1. Disparities in incomes and savings by households with different age structures and CHE status

**
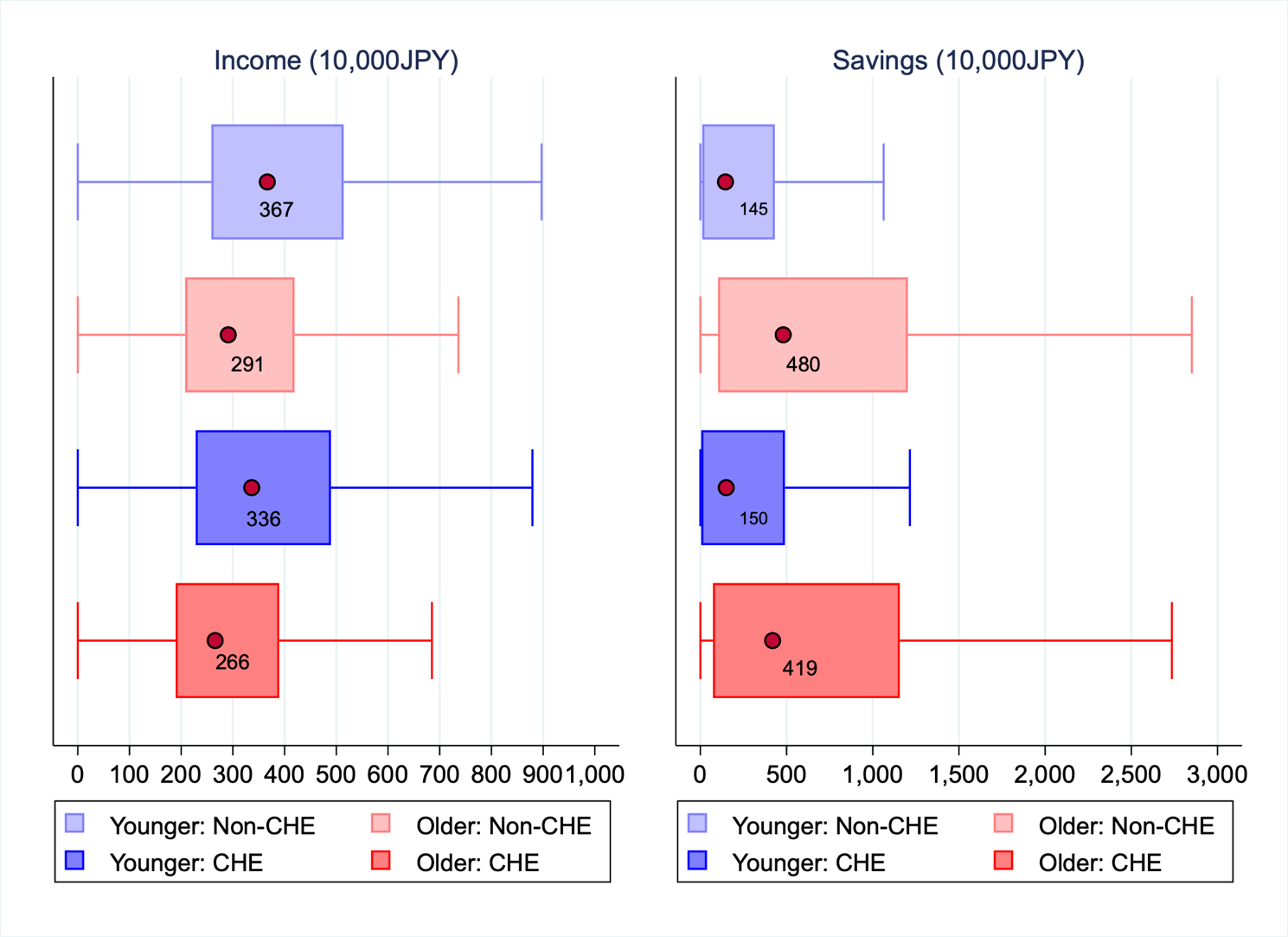
**

Note: 'Older' represents household with members aged 65 or older, whilst 'Younger' denotes households only with members aged < 65 years; Both income and savings are equalised by household size; Sample sizes of each category from the top are: n=42,351; n=17,409; n=3,300; n=3,152 (person-year observations, 2004-2020); Points and values in the boxes represent median values.

**References for Supplementary material**

1. Xu K, Evans DB, Kawabata K, Zeramdini R, Klavus J, Murray CJL. Household catastrophic health expenditure: a multicountry analysis. The Lancet. 2003;362(9378):111-7.

2. Xu K, Evans DB, Carrin G, Aguilar-Rivera AM, Musgrove P, Evans T. Protecting households from catastrophic health spending. Health Aff (Millwood). 2007;26(4):972-83. Epub 2007/07/17.

3. Wagstaff A, Flores G, Hsu J, Smitz M-F, Chepynoga K, Buisman LR, et al. Progress on catastrophic health spending in 133 countries: a retrospective observational study. The Lancet Global Health. 2018;6(2):e169-e79.

4. United Nations Statistics Division. E-Handbook on SDG Indicators: Indicator 3.8.2. 2018 [cited 2021 29 November]; Available from: <https://unstats.un.org/wiki/display/SDGeHandbook/Indicator+3.8.2>.

5. World Health Organization, World Bank. Tracking Universal Health Coverage: 2023 Global Monitoring Report. Geneva: World Health Organization and International Bank for Reconstruction and Development / The World Bank; 2023.

6. Wagstaff A, Flores G, Smitz M-F, Hsu J, Chepynoga K, Eozenou P. Progress on impoverishing health spending in 122 countries: a retrospective observational study. The Lancet Global Health. 2018;6(2):e180-e92.

7. van Dongen SI, van Straaten B, Wolf J, Onwuteaka-Philipsen BD, van der Heide A, Rietjens JAC, et al. Self-reported health, healthcare service use and health-related needs: A comparison of older and younger homeless people. Health Soc Care Community. 2019;27(4):e379-e88. Epub 20190425.

8. Allin S, Masseria C, Mossialos E. Equity in health care use among older people in the UK: an analysis of panel data. Appl Econ. 2011;43(18):2229-39.

9. Veugelers PJ, Yip AM. Socioeconomic disparities in health care use: Does universal coverage reduce inequalities in health? J Epidemiol Community Health. 2003;57(6):424-8.

10. Okamoto S, Komamura K. Towards universal health coverage in the context of population ageing: a narrative review on the implications from the long-term care system in Japan. Arch Public Health. 2022;80(1):210. Epub 20220921.

11. Gibson G, Grignon M, Hurley J, Wang L. Here comes the SUN: Self-assessed unmet need, worsening health outcomes, and health care inequity. Health Econ. 2019;28(6):727-35. Epub 2019/04/26.

12. OECD. Health at a Glance 2011: OECD Indicators. OECD Publishing; 2011.

13. Yamada T, Chen CC, Murata C, Hirai H, Ojima T, Kondo K, et al. Access disparity and health inequality of the elderly: unmet needs and delayed healthcare. Int J Environ Res Public Health. 2015;12(2):1745-72. Epub 20150203.

14. Bataineh H, Devlin RA, Barham V. Unmet health care and health care utilization. Health Econ. 2019;28(4):529-42. Epub 20190128.

15. Herr M, Arvieu JJ, Aegerter P, Robine JM, Ankri J. Unmet health care needs of older people: prevalence and predictors in a French cross-sectional survey. Eur J Public Health. 2014;24(5):808-13. Epub 20131127.

16. Rahman MM, Rosenberg M, Flores G, Parsell N, Akter S, Alam MA, et al. A systematic review and meta-analysis of unmet needs for healthcare and long-term care among older people. Preprint (Posted on 08 April, 2022). 2022.

17. Rosenberg M, Kowal P, Rahman MM, Okamoto S, Barber SL, Tangcharoensathien V. Better data on unmet healthcare need can strengthen global monitoring of universal health coverage. BMJ. 2023;382:e075476. Epub 20230905.

18. Mooney G. Is it not time for health economists to rethink equity and access? Health Economics, Policy and Law. 2009;4(2):209-21. Epub 2009/04/01.

19. Ju YJ, Kim TH, Han KT, Lee HJ, Kim W, Ah Lee S, et al. Association between unmet healthcare needs and health-related quality of life: a longitudinal study. Eur J Public Health. 2017;27(4):631-7.

20. World Health Organization, World Bank. Tracking universal health coverage: 2021 global monitoring report. 2021.
